# Sequential Objective Structured Clinical Examination based on item response theory in Iran

**DOI:** 10.3352/jeehp.2017.14.19

**Published:** 2017-09-08

**Authors:** Sara Mortaz Hejri, Mohammad Jalili

**Affiliations:** 1 Health Professions Education Research Center, Tehran University of Medical Sciences, Tehran, Iran; 2 Department of Emergency Medicine, Tehran University of Medical Sciences, Tehran, Iran; 3 Department of Medical Education, Tehran University of Medical Sciences, Tehran, Iran; Hallym University, Korea

**Keywords:** Sequential, Objective structured clinical examination, Item response theory, Cost

## Abstract

**Purpose:**

In a sequential objective structured clinical examination (OSCE), all students initially take a short screening OSCE. Examinees who pass are excused from further testing, but an additional OSCE is administered to the remaining examinees. Previous investigations of sequential OSCE were based on classical test theory. We aimed to design and evaluate screening OSCEs based on item response theory (IRT).

**Methods:**

We carried out a retrospective observational study. At each station of a 10-station OSCE, the students’ performance was graded on a Likert-type scale. Since the data were polytomous, the difficulty parameters, discrimination parameters, and students’ ability were calculated using a graded response model. To design several screening OSCEs, we identified the 5 most difficult stations and the 5 most discriminative ones. For each test, 5, 4, or 3 stations were selected. Normal and stringent cut-scores were defined for each test. We compared the results of each of the 12 screening OSCEs to the main OSCE and calculated the positive and negative predictive values (PPV and NPV), as well as the exam cost.

**Results:**

A total of 253 students (95.1%) passed the main OSCE, while 72.6% to 94.4% of examinees passed the screening tests. The PPV values ranged from 0.98 to 1.00, and the NPV values ranged from 0.18 to 0.59. Two tests effectively predicted the results of the main exam, resulting in financial savings of 34% to 40%.

**Conclusion:**

If stations with the highest IRT-based discrimination values and stringent cut-scores are utilized in the screening test, sequential OSCE can be an efficient and convenient way to conduct an OSCE.

## Introduction

Objective structured clinical examinations (OSCEs), especially if composed of a large number of stations and administered to a significant number of candidates, consume an excessive amount of resources. Considering the cost of paying raters, staff, and standardized patients, and given current economic imperatives, feasibility is regarded as a serious hindrance to OSCE administration. As Harden [1] pointed out in one of his recent papers, “the most frequently cited argument against the use of the OSCE relates to cost”. Meanwhile, it has been suggested that the cost of the OSCE can be reduced by using a sequential design [1,2], which can be considered a simple form of adaptive testing. In a sequential OSCE, all students initially take a screening OSCE with a reduced number of stations. Examinees who perform well relative to the cut-score are excused from further testing, but an additional OSCE is administered to the remaining examinees, who are below the pass-fail boundary.

Several studies have explored the use of sequential OSCE. In most of them, a sequential test was not actually administered; instead, a hypothetical testing situation was considered, in which several screening tests were designed based on retrospective data and their results were compared to the main OSCE decisions [3-5]. Although it seems that a substantial reduction in testing time and resources can be achieved through this approach, careful consideration is required when designing sequential OSCEs. The screening OSCE, which is a relatively short and therefore a fairly unreliable test, should accurately and efficiently identify students at the upper ability levels. First, this means that most students should perform well relative to the cutscore of the screening test. If the pass rate is not high enough, a substantial number of students have to take the second OSCE, and the use of the sequential model would not be beneficial from the perspective of resources. Second, candidates with a passing result should be truly competent, and within this group of students, there should be no false positives, which correspond to examinees who are actually incompetent. Likewise, in the group of failing students, although some doubt persists and it is probable that both competent and incompetent students will be included, the number of false negatives (truly competent students who fail the screening test) should be kept reasonably low.

To achieve these goals, one should answer these 3 questions when designing a screening test: what is the optimal number of required stations, how should we select the stations, and what is the best cutscore for the screening OSCE? While previous investigations have tried to answer these questions to some extent [3-5], they all faced an important limitation when trying to answer the second question. The selection of stations in these studies was based on stations’ difficulty and discrimination indices calculated under the premises of classical test theory, which suffers from sample-dependency. In other words, the difficulty and discrimination values for a given test item do not just represent the targeted content characteristics, but are also dependent on the ability of students who take the exam. Thus, the difficulty and discrimination values would not remain constant for a different group of examinees, meaning that they are not generalizable to other circumstances. To resolve this problem, it has been suggested that item response theory (IRT) should be used to estimate item parameters as a function of the characteristics of the item, as well as the underlying ability of the examinees [6]. Although IRT has been widely applied in other settings, such as item bank development, computer adaptive testing, and test equating, we could not find any studies using this approach for designing a sequential OSCE. In the current study, we aimed to design several hypothetical screening OSCEs based on IRT parameters and to evaluate their performance.

## Methods

### Study design

We carried out a retrospective observational study.

### Materials and subjects

Data for this study were gathered from 10 stations of a pre-internship OSCE at Tehran University of Medical Sciences. At each station, a trained professional rater observed the students’ performance and graded them, according to a scoring rubric, on a Likert-type scale (1: fail to 5: competent). Since the data were polytomous, the difficulty parameters, discrimination parameters, and students’ ability (theta from −3 to +3), were calculated by applying a graded response model. Considering the difficulty parameter, 4 category parameters were estimated for each station, due to the presence of 5 boundaries for responses. Consequently, we considered the mean of these 4 difficulty parameters to be the difficulty of the station. Item characteristic curves (ICCs) were drawn for all stations, and a test information curve was generated for the total test. The main OSCE minimum pass level (MPL) was decided arbitrarily, and an examinee passed the test if his/her theta was greater than or equal to zero.

We decided that each screening OSCE would be shortened to onethird to half of the main OSCE. Thus, 5, 4, or 3 stations were selected for each screening test. To choose the stations, we considered the stations’ parameters, and identified the 5 most difficult stations and the 5 most discriminative ones. One screening OSCE, for instance, was composed of the 3 most difficult stations, while another screening OSCE had the 4 stations with the highest discrimination values. The theta value for each student (−3 to +3) was calculated for each of the 6 screening tests. Similar to the main OSCE, we applied an arbitrary MPL for each test. However, 2 forms of MPL were defined: a normal MPL (theta greater than zero), and a stringent MPL (theta greater than 0.2). In total, considering the aforementioned 3 factors (stations’ parameters, number of stations, and MPLs), we designed 12 screening tests (2 × 3× 2). We compared the results of each screening OSCE to the main OSCE according to a 2 × 2 table, and calculated the corresponding positive and negative predictive values (PPV and NPV). For the sequential design to be a good substitute for the current routine practice, the screening OSCE should predict the results of the main OSCE correctly (i.e., with no false positives) and in an efficient way (a reasonable NPV and pass rate). Hence, we decided to consider a sequential design to be desirable if the PPV, NPV, and pass rate were 1.0, ≥ 0.25, and ≥ 50%, respectively.

The cost for each student in the total test, in our setting, included *$*5 per station plus *$*9 as a fixed charge [(10 stations×*$*5)+*$*9]. Thus, the cost of the main OSCE (consisting of 10 stations) was calculated as follows: c= all students× [(10 stations× *$*5)+*$*9]. If a sequential design were used, the total cost would be the cost of conducting the screening test {266 students×[(number of the screening stations× *$*5) +*$*9]} in addition to the cost of conducting the second OSCE {number of failed students×[(number of the remaining stations×*$*5)+*$*9]}.

Hence, the benefit of applying the sequential design could be estimated by subtracting the cost of the sequential design from the cost of the main OSCE.

### Statistics

The difficulty and discrimination parameters were calculated by applying a graded response model using Multilog ver. 7.0 (Scientific Software International, Lincolnwood, IL, USA). The PPV, pass rate, and NPV of each screening OSCE and their 95% confidence intervals were computed from a 2 × 2 contingency table.

### Ethical approval

The study proposal was approved by the Postgraduate Studies Council in School of Medicine, Tehran University of Medical Sciences (No. 8921486007) after receiving informed consent from subjects, and since it was to be conducted on existing anonymous data from previous years, the study was exempted from ethical review.

## Results

**Fig. 1.**
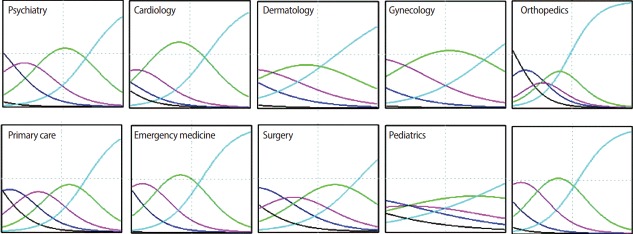
The item characteristic curves for each of the 10 stations in the main objective structured clinical examination.

**Fig. 2.**
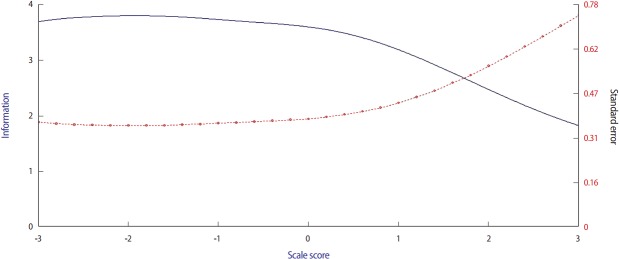
The test information curve (upper curve) and standard error curve (lower curve) of the main objective structured clinical examination.

Data from 266 examinees were included in the study. Raw data were available from Supplement 1. The station difficulty and discrimination parameters are shown in Table 1. The ranges of the difficulty parameter and discrimination parameter were −2.96 to −0.89 and 0.57 to 1.38, respectively. The 5 most difficult stations were primary care, surgery, orthopedics, pediatrics, and psychiatry. The 5 most discriminative stations were orthopedics, internal medicine, emergency medicine, primary care, and psychiatry. The ICCs for each station can be seen in Fig. 1. Additionally, the test information curve is presented in Fig. 2. Considering students’ ability in the whole OSCE, it was found that the minimum and maximum theta were −0.61 and 1.96. The average theta was 0.84, with a standard deviation of 0.48. Overall, 253 students (95.1%) had a theta greater than zero and passed the main OSCE.

As can be seen in Table 2, 12 screening tests were designed according to different number of stations (3, 4, and 5), 2 type of parameters (difficulty and discrimination), and 2 forms of MPL (normal and stringent). Students’ abilities (theta) are shown in Table 2. The lowest theta (−1.34) was obtained in the screening OSCE with 4 difficult stations (Dif-4). The highest theta (1.89) was achieved in 2 distinct screening OSCEs (Dif-5 and Dis-5) (Table 2). When the normal MPL was used, 86.8% to 94.4% of examinees passed the screening test. According to the stringent MPL, the pass rate ranged from 72.6% to 85.3% (Table 2). Moreover, the PPV values ranged from 0.98 to 1.00, and the NPV values ranged from 0.18 to 0.59.

While most of the screening tests had a pass rate greater than 75% and 8 screening tests had an acceptable NPV, 4 screening OSCEs detected no false-positive results. Only 2 tests were identified as desirable screening OSCEs and effectively predicted the results of the main exam. In both, the stations were selected based on the discrimination parameter, and the stringent MPL was applied. One of them had 4 stations, and the other was a 5-station OSCE.

The cost of the main OSCE for all students was *$*15,694. The cost of the screening OSCEs coded Dis-5-S and Dis-4-S were *$*9,044 and *$*7,714, respectively. Hence, if the sequential design had been applied by using these 2 screening tests, the total cost of the screening test plus the second exam would have been *$*10,370 and *$*9,352, respectively. Thus, in comparison to the main OSCE, *$*5,324 (33.9%) or *$*6,342 (40.4%) would have been saved, respectively.

## Discussion

In this study, we considered and evaluated different screening OSCE models to identify an optimal sequential model. Twelve screening tests were designed considering 3 factors: a different number of stations, the use of the difficulty or discrimination parameter, and 2 forms of MPL. After comparing the results of the screening OSCEs with the main OSCE, it was found that in the 2 favorable sequential OSCEs, stations with a high discrimination parameter were selected and a stringent MPL was applied. These screening OSCEs predicted the main exam results accurately (with no false positives) and cost-effectively (savings over 30%).

Several studies have designed screening OSCEs in different ways and have reached different results regarding the most appropriate type of screening test. For instance, Culliver et al. [7,8] concluded that selecting one-third of the main OSCE stations and using stringent cut-scores would yield acceptable decisions. The study by Rothman et al. [9] showed that using 10 stations chosen randomly from the main 20-station OSCE and applying a relatively stringent cutoff score could lead to an accurate pass-fail classification of students, as well as a significant savings in OSCE costs. Pell et al. [3] introduced a sequential model of 12 stations in the first test and 12 stations in the second test to maximize cost savings. In that study, 13.5% of students (n= 228) failed the first test, and compared to the results of the 24-station OSCE, 1 student (0.4%) and 9 students (3.9%) were identified as false positive and false negative results, respectively. The authors concluded that the savings facilitated by their sequential model amounted to approximately GBP 29,000 [3].

In the above studies, the investigators conducted post-hoc analyses of OSCE data. In contrast, 2 studies have analyzed the administration of real sequential OSCEs. Since the students who passed the screening tests did not take the second OSCE, these 2 studies could not report false positive rates. However, they reported useful data on OSCE costs. Smee and colleagues ran a sequential OSCE for the Medical Council of Canada in 1997. In 14 exam centers across Canada, 1,952 candidates took the screening OSCE, composed of 10 stations with high item-total correlation values. Fewer than 40% of students (n=546) failed the screening test and took the second OSCE with 10 stations. Considering the 20-station OSCE as a whole, it was found that 1.7% of the cohort failed the exam. Comparing costs to the previous year, the authors reported that about *$*350,000 was saved, mainly by reducing the number of examiners and standard patients [10]. In another study by Cookson et al. [11], a sequential test was administered to 127 students at the Hull York School of Medicine, and approximately GBP 30,000 was reported to have been saved.

It should be noted that none of the previous studies on sequential OSCE used the psychometric properties extracted from IRT. Few studies have investigated OSCEs based on IRT models, partly because IRT-based analyses require a large sample size (a large number of test-takers) and the nature of polytomous data (checklist or global scores) requires more complex analytical models (compared to multiple-choice questions). Moreover, most of these studies only calculated the difficulty parameter based on the Rasch model. In contrast, in our study, in addition to the difficulty parameter, the discrimination parameter based on the graded response model was also considered.

Our study faces some limitations. First, to determine the prediction accuracy, the main exam was treated as the gold standard. One might argue that there may be problems with the main OSCE. Another limitation of this study, as with many similar studies in this field, is its retrospective design. In other words, sequential testing has not really been implemented; the screening tests were designed and evaluated hypothetically based on an analysis of pre-existing data. It is suggested that future studies design sequential OSCE based on our proposed model and examine its cost-effectiveness, as well as the practical problems that might occur during the process.

## Figures and Tables

**Table 1.** The difficulty and discrimination parameters of the stations, according to the graded response model

Station no.	Station name	Difficulty parameter in terms of Likert levels				Difficulty parameter	Discrimination parameter
		Level 1	Level 2	Level 3	Level 4		
1	Psychiatry	-5.97	-2.82	-1.11	1.31	-1.71^a)^	1.03^b)^
2	Cardiology	-4.67	-3.43	-1.97	0.87	-1.84	1.01
3	Dermatology	-9.21	-4.78	-2.10	0.91	-3.03	0.57
4	Gynecology	-9.88	-5.34	-1.87	-2.29	-2.96	0.58
5	Orthopedics	-2.90	-1.83	-1.14	-0.12	-1.19^a)^	1.38^b)^
6	Primary care	-9.39	-1.88	-0.46	1.24	-0.89^a)^	1.13^b)^
7	Emergency medicine	-7.61	-3.36	-1.63	0.49	-2.42	1.14^b)^
8	Surgery	-4.38	-2.10	-0.38	2.05	-0.96^a)^	0.78
9	Pediatrics	-7.36	-3.32	-0.49	3.48	-1.53^a)^	0.36
10	Internal medicine	-5.39	-3.51	-1.71	0.27	-2.06	1.17^b)^

a) Stations selected based on the difficulty parameter.

b) Stations selected based on the discrimination parameter.

**Table 2.** Screening objective structured clinical examinations

Code	Parameter	No. of stations	MPL	Included stations	Students’ ability (theta)	Pass rate (%)	Negative predictive value	Positive predictive value
Dif-5-N	Difficulty	5	Normal^a)^	Primary care, surgery, orthopedics, pediatrics, psychiatry	-1.02 to 1.89	234 (88.0)	0.34	0.99
Dif-5-S	Difficulty	5	Stringent^b)^	Primary care, surgery, orthopedics, pediatrics, psychiatry	-1.02 to 1.89	211 (79.3)	0.24	1.00
Dif-4-N	Difficulty	4	Normal	Primary care, surgery, orthopedics, pediatrics	-1.34 to 1.70	234 (88.0)	0.34	0.98
Dif-4-S	Difficulty	4	Stringent	Primary care, surgery, orthopedics, pediatrics	-1.34 to 1.70	193 (72.6)	0.18	1.00
Dif-3-N	Difficulty	3	Normal	Primary care, surgery, orthopedics	-1.13 to 1.13	231 (86.8)	0.29	0.98
Dif-3-S	Difficulty	3	Stringent	Primary care, surgery, orthopedics	-1.13 to 1.13	207 (77.8)	0.18	0.99
Dis-5-N	Discrimination	5	Normal	Orthopedics, internal medicine, emergency medicine, primary care, psychiatry	-0.54 to 1.89	249 (93.6)	0.59	0.98
Dis-5-S	Discrimination	5	Stringent	Orthopedics, internal medicine, emergency medicine, primary care, psychiatry	-0.54 to 1.89	227 (85.3)	0.33	1.00
Dis-4-N	Discrimination	4	Normal	Orthopedics, internal medicine, emergency medicine, primary care	-0.55 to 1.70	251 (94.4)	0.53	0.98
Dis-4-S	Discrimination	4	Stringent	Orthopedics, internal medicine, emergency medicine, primary care	-0.55 to 1.70	224 (84.2)	0.31	1.00
Dis-3-N	Discrimination	3	Normal	Orthopedics, internal medicine, emergency medicine	-1.13 to 1.13	231 (86.8)	0.29	0.98
Dis-3-S	Discrimination	3	Stringent	Orthopedics, internal medicine, emergency medicine	-1.13 to 1.13	204 (76.7)	0.18	0.99

MPL, minimum pass level.

a) Normal MPL: theta ≥0.

b) Stringent MPL: theta ≥0.2.

## Supplementary materials

Supplement 1. Data file is available from https://dataverse.harvard.edu/dataset.xhtml?persistentId=doi%3A10.7910%2FDVN%2FVG1AJW

Supplement 2. Audio recording of the abstract
